# Spinal excitability is enhanced by transcranial magnetic stimulation of the motor cortex in children and adolescents

**DOI:** 10.1016/j.cnp.2025.06.009

**Published:** 2025-07-06

**Authors:** Essi J. Marttinen Rossi, Päivi Nevalainen, Jussi Toppila, Helena Mäenpää, Jessica Guzmán-López, Harri Piitulainen, Leena Lauronen

**Affiliations:** a Motion Laboratory, New Children’s Hospital, Helsinki University Hospital and University of Helsinki, Helsinki, Finland; b BioMag Laboratory, HUS Diagnostic Center, Helsinki University Hospital and University of Helsinki, Helsinki, Finland; c Pediatric Research Center, New Children’s Hospital, Helsinki University and Helsinki University Hospital, Helsinki, Finland; d Clinical Neurophysiology, HUS Diagnostic Center, Helsinki University Hospital and University of Helsinki, Helsinki, Finland; e Child Neurology, New Children’s Hospital, Helsinki University Hospital and University of Helsinki, Helsinki, Finland; f Faculty of Medicine, National Autonomous University of Mexico (UNAM), Mexico City, Mexico; g Faculty of Sport and Health Sciences, University of Jyväskylä, Finland

**Keywords:** H-reflex conditioning, Transcranial magnetic stimulation, Spinal cord excitability, Corticospinal pathway, Facilitation, Children

## Abstract

**Objective:**

To investigate the modulation of spinal excitability to sub-motor threshold transcranial magnetic stimulation (TMS) in healthy children and adolescents.

**Methods:**

We paired sub-motor threshold TMS (conditioning) with electrical tibial nerve stimulation (test) to assess changes in the soleus H-reflex at eleven conditioning-test (C-T) intervals. Eleven participants (7.5–16.2 years) were studied under three conditions: rest, voluntary agonist activation (ankle plantar flexion), and voluntary antagonist activation (ankle dorsiflexion).

**Results:**

TMS-induced supraspinal volley facilitated the soleus H-reflex amplitude significantly during rest and agonist activation, but not during antagonist activation. The facilitation of H-reflex was similar during rest and agonist activation between C-T intervals from –2 ms (i.e. peripheral stimulation before cortical) to +18 ms (i.e. cortical stimulation before peripheral). Specific to rest condition, a later facilitation occurred at C-T intervals of approximately +60 ms.

**Conclusions:**

Spinal excitability modulation in children and adolescents showed similarities to earlier evidence in adults, with the distinction that, unlike in the adults, agonist activity did not further enhance the supraspinal facilitation of the H-reflex compared to the rest condition in children.

**Significance:**

These results provide insight into the maturation of motor control and regulation of spinal excitability, offering possibilities to identify typical and atypical developmental trajectories.

## Highlights

• TMS induced supraspinal input changes the spinal excitability in children and adolescents.
• TMS enhanced spinal excitability at rest and during agonist, but not antagonist muscle activation, similarly as in adults.
• Unlike in adults, agonist activity did not potentiate the faciliatory effect of TMS.

## 1 Introduction

Spinal cord motor excitability is essential for modulating and refining posture and movements in humans, influenced not only by cortical control (efference), but also significantly by somatosensory feedback (afference) generated from posture and movement (Abbruzzese et al., 1996, Bove et al., 2006, Goulart et al., 2000). Supraspinal inhibitory control over spinal networks gradually strengthens as the nervous system matures, maturation including for example the myelination of the corticospinal tract speeding up conduction times that reach adult levels in the second decade of life (Koh and Eyre, 1988). Given these ongoing developmental changes, brain control over spinal motor networks may function differently in children and adults. However, the effect of supraspinal input on spinal excitability in children has not been explored.

Adult studies have examined the impact of supraspinal input on spinal excitability using transcranial magnetic stimulation (TMS) (Goulart et al., 2000, Petersen et al., 2003). Pairing a sub-motor threshold TMS pulse with peripheral nerve stimulation (PNS) at several intervals in a so called “conditioning-test” (C-T) paradigm, enables assessment of spinal excitability over time using the soleus Hoffman (H) reflex as an indicator (Nielsen et al., 1993, Valls-Sole et al., 1994, Guzmán-López et al., 2012, Guzmán-López et al., 2015; Taube et al., 2017, Andrews et al., 2020, Xu et al., 2022). In adults, TMS elicits a short-latency facilitation of the H-reflex at C-T intervals –5 to –3 ms (the negative interval indicating that TMS follows PNS), followed by an inhibition at C-T intervals from –3 to –1 ms, which are thought to be mediated by inputs from direct corticomotoneuronal and reciprocal pathways, respectively (Nielsen and Petersen, 1995b, Andrews et al., 2020). A more pronounced long-latency facilitation of the H-reflex has been observed in adults at C-T intervals of +10 to +20 ms (TMS precedes PNS) (Valls-Sole et al., 1994, Goulart et al., 2000, Costa et al., 2011, Guzmán-López et al., 2012, Andrews et al., 2020), likely due to the summation of excitatory post-synaptic potentials (EPSPs) in the lower motoneurons (Goulart et al., 2000, Serranová et al., 2008, Costa et al., 2011) and a reduction in presynaptic inhibition of the primary sensory afferents (Ia) (Rudomin, 1990, Valls-Sole et al., 1994; Guzmán-López et al., 2012). Once the excitatory effects fade and presynaptic inhibition is restored, supraspinal stimulation no longer modulates the H-reflex in adults, typically from C-T intervals +40 to +60 ms (Costa et al., 2011, Goulart et al., 2000, Guzmán-López et al., 2012, Guzmán-López et al., 2015). This period of no modulation is followed by a delayed re-facilitation of the motoneuron pool from C-T intervals +70 to +120 ms. Additionally, in adults, voluntary agonist muscle activation further enhances the facilitatory effect of cortical volleys on spinal networks (Nielsen and Petersen, 1995b, Lazzaro et al., 1998; Guzmán-López et al., 2015), while antagonist activation diminishes the facilitatory effect (Crone and Nielsen, 1994; Guzmán-López et al., 2015; Lazzaro et al., 1998).

To our knowledge, it is unclear whether sub-motor threshold TMS induced supraspinal volleys from the motor cortex, acting on spinal networks, modulate spinal excitability in the developing central nervous system, and if this modulation aligns with previous evidence on the mature system. Therefore, we examined how activation by supraspinal volleys, induced by sub-motor threshold TMS, affects spinal excitability – measured through H-reflex amplitude – in children and adolescents, and how voluntary agonist and antagonist muscle activation influences this pattern of spinal excitability.

## 2 Methods

### 2.1 Participants

We recruited 11 healthy children (2 participants were 7–9 years, 2 were 10–12 years, 3 were 13–14 years, and 4 were 15–16 years, mean 13.4, standard deviation ± 2.9 years), with no known motor or neurological conditions, from the children of hospital staff members and friends. We received informed written consent from each child and parent. The study was approved by the Women’s, Children’s and Psychiatric Ethics Committee of the Helsinki University Hospital (Reference No. 197/13/03/2012).

### 2.2 Imaging

All participants underwent brain magnetic resonance imaging (MRI) with a 3 T scanner. The T1 images were reconstructed into 3D models of the head and the brain, which were utilized for eXimia 3.2 navigation brain system TMS (NexStim Ltd, Finland). Two participants had coincidental finds in the MRI: one had a Chiari I-type finding, and the other had an arachnoid cyst in the posterior fossa. We assessed that these findings would not impact the study’s objectives and, as such, were not grounds for excluding the participants from the study.

### 2.3 General procedure

This study examined the effect of sub-motor threshold TMS, delivered to the left primary motor cortex, on the contralateral soleus H-reflex under three conditions: rest, voluntary agonist activation (plantar flexion), and voluntary antagonist activation (dorsiflexion).

*Plantar and dorsiflexion forces:* We measured voluntary contraction forces of plantar and dorsiflexor muscles using a custom-made aluminum system with an embedded force transducer (Tedea Huntleigh, Model 1042, Glostrup, Denmark). Initially, we assessed each participant’s maximal voluntary plantar and dorsiflexion forces. During the plantar and dorsiflexion conditions, participants maintained a steady contraction at 15 % of their maximal voluntary contraction, guided by visual feedback from the force transducer.

**Fig. 1:**
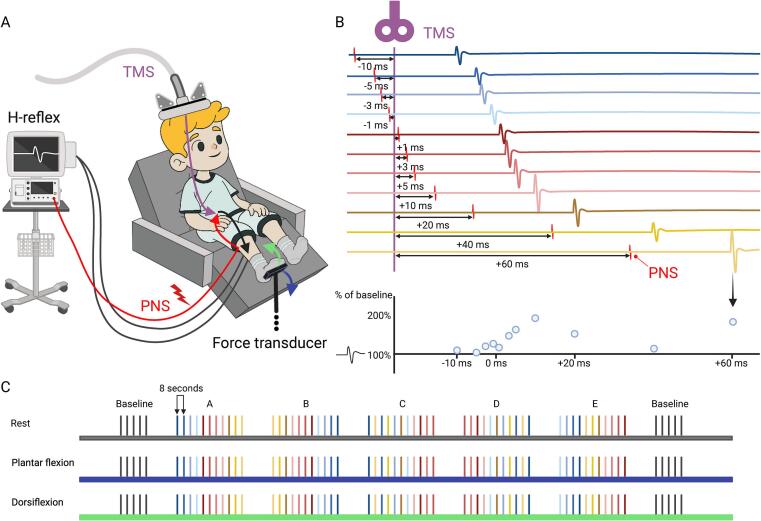
Study protocol A: Sub-motor threshold neuronavigated transcranial magnetic stimulation (nTMS) was applied to the left motor cortex and paired with peripheral nerve stimulation (PNS) of the right tibial nerve. Motor responses were recorded from the soleus muscle as Hoffman (H) reflexes during rest, agonist activation (blue arrow) and antagonist activation (green arrow) conditions. Muscle activation was maintained at 15 % of maximal voluntary contraction using a custom-built force transducer and visual feedback. B: nTMS and PNS were paired at eleven conditioning-test (C-T) intervals ranging from –10 to +60 ms. Negative values indicate that PNS precedes TMS, while positive values indicate that TMS precedes PNS. The graph at the bottom of section B (light blue circles) illustrates the modulatory effects of TMS on H-reflex amplitudes. C: The eleven C-T intervals (color-coded lines, each line representing one paired stimulation with colors matching to figure B) were presented semi-randomly across five sessions (A-E). These five sessions were preceded and followed by baseline sessions without TMS. An eight second washout period followed each paired stimulation. Measurements were carried out at rest and during agonist and antagonist activation. Created in BioRender. Nevalainen, P. (2025) https://BioRender.com/w25c170. (For interpretation of the references to color in this figure legend, the reader is referred to the web version of this article.)

*Measurement position:*Fig. 1A illustrates how, throughout the study, participants were comfortably seated in an adjustable reclining chair with their right knee supported at a 20° flexion angle using pillows and their right ankle positioned at 20° extension. The foot was secured to a metal plate attached to the force transducer to minimize movement during measurements. Knee and ankle angles were continuously visually monitored in each session.

*H-reflex measurement:* We elicited the H-reflex by electrically stimulating the tibial nerve at the posterior popliteal fossa with a 1 ms bipolar stimulus using the Keypoint portable 4-channel device (Keypoint.net). After determining the maximal H-reflex amplitude, the tibial nerve stimulation intensity was lowered to produce an H-reflex corresponding to 50 % of its maximum amplitude. This allowed for the response to be either facilitated or suppressed in the subsequent sessions. In the antagonist activation condition, the stimulus intensity had to be increased compared to the rest and agonist activation conditions to achieve the 50 % target. To avoid exposing the participants to strong, unpleasant stimuli at the beginning of the experiment, we measured the maximum M−wave at the end of the study. Thus, we did not to use a percentage of the maximum M−wave, typically 20–25 % when the H-reflex is most sensitive to facilitation (Crone et al., 1990), but we retrospectively calculated that 50 % of the maximum H-reflex amplitude corresponded to an average of 28.9 % of the maximum M−wave amplitude.

*TMS measurement:* TMS was delivered as a monopolar square wave single pulse using a figure-of-8 coil (Focal MonoPulse, Ref.no: 201514P-C) with a Nexstim eXimia (NBS3) navigated TMS device. The coil was positioned over the vertex to activate the leg area of the cortex. The brain location and coil orientation that produced the strongest soleus motor evoked potentials (MEPs) were identified and locked in place on the MRI. Surface EMG electrodes were used to record soleus muscle activity. The motor threshold for the soleus MEP was defined as the lowest stimulus intensity producing a ≥ 30 µV response at 3 out of 6 trials, and the TMS intensity was set to 90 % of this resting motor threshold (RMT).

*C-T protocol:*Fig. 1B demonstrates the timing of the stimulations. Sub-motor threshold TMS (conditioning stimulus) was paired with electrical tibial nerve stimulation (test stimulus) producing a soleus H-reflex. H-reflexes were recorded at conditioning-test (C-T) intervals of –10, –5, –3, –1, +1, +3, +5, +10, +20, +40 and +60 ms, where negative intervals indicated that PNS preceded TMS, and positive intervals indicated that TMS preceded PNS. Fig. 1C illustrates how sequences of paired stimuli were programmed into SPIKE 2 (CED, Cambridge Electronic Design Limited) and delivered in a semi-random order across five sessions, with baseline sessions (no TMS) conducted before and after the main sessions. Each paired stimulation was separated by an eight second wash-out period to eliminate any residual effects from the previous response and return responses back to baseline.

### 2.4 Data analysis

The amplitudes of the soleus H-reflex were measured as the peak-to-peak value of the response, with the onset defined as the point where the curve deviated from baseline. H-reflexes were averaged according to the C-T intervals, and peak absolute amplitudes were compared to condition-specific baselines, calculated as the average of the pre- and post-session baseline stimuli (excluding the two most extreme values in each set).

The latencies for MEPs (26–32.5 ms) and H-reflexes (20–31.5 ms) varied among participants with the H-reflex having a slightly shorter latency than the MEP, difference ranging from 0.5 to 6 ms, in all but one participant in whom the MEP latency was 0.5 ms shorter than the H-reflex latency. These latency variations in conduction times meant that we could not assume that simultaneously administered central and peripheral stimulations resulted in supraspinal and peripheral volleys colliding in the spinal cord. To account for individual differences, we retrospectively corrected the latency difference by subtracting the H-reflex latency from the MEP latency for each participant, thus calculating participant-specific C-T intervals (corrected C-T interval = original C-T interval – (MEP latency – H-reflex latency)). After applying the latency correction, we could assume that the central and peripheral volleys activate the spinal neurons simultaneously at C-T interval 0 ms, and that negative C-T intervals indicate the time by which the supraspinal volley follows the peripheral volley, and positive C-T intervals the time by which the supraspinal volley precedes the peripheral volley in the spinal cord. Due to our latency correction, the C-T intervals varied between participants, and for group analysis responses had to be temporally grouped and averaged to estimate the original C-T intervals as illustrated in Table 1. This average was used as the corrected C-T interval.

**Table 1:** The original conditioning-test (C-T) intervals represent the time difference (ms) by which the cortical and peripheral stimulations are administered. In the corrected C-T intervals, individual participant’s variations in the latencies of supraspinal and peripheral volleys are accounted for, after which the two volleys are presumed to activate the spinal neurons simultaneously at C-T interval 0. Negative values indicate the duration by which the peripheral volley precedes the cortical one and the positive values by which the cortical precedes the peripheral one. After applying the latency-correction, responses were temporally grouped to closely align the corrected and original C-T intervals, minimizing the risk of missing data points. The average latency was calculated from these interval ranges and used in the analysis as the corrected C-T interval.

Original C-T interval (ms)	Corrected C-T interval range (ms)	Corrected C-T interval average (ms)
−10	–16 to –7	∼ –10
−5	–6.5 to –4.5	∼ –6
−3	–4 to –2.5	∼ –4
−1	–2 to –0.5	∼ –2
+1	0 to +3.5	∼ +1
+3		
+5	+4 to +10.5	∼ +7
+10		
+20	+14 to +20.5	∼ +18
+40	+34 to +40.5	∼ +38
+60	+54 to +60.5	∼ +58

### 2.5 Statistics

After normality testing of the data (Shapiro-Wilk test), we used the Friedman test for within condition comparisons and comparisons across conditions. Wilcoxon’s Signed Rank test with Bonferroni’s correction was used for post hoc testing. When comparing modulatory effects across the three conditions, H-reflex amplitudes were presented as percentages of their condition-specific baselines. Spearman’s Rho and Mann Whitney U tests were used when correlating age with the modulatory effects observed in the H-reflex. SPSS version 28 was used in the analyses and statistical significance was defined at P < 0.05.

## 3 Results

### 3.1 Effect of the conditioning TMS pulse on the H-reflex amplitude at different conditioning-test intervals

**Fig. 2:**
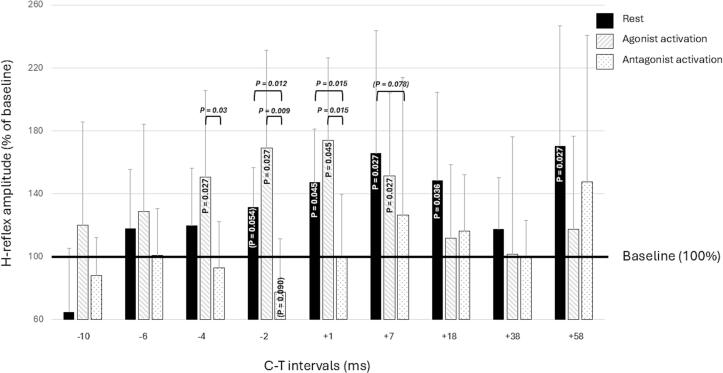
Supraspinal modulation of the soleus H-reflex amplitude at nine C-T intervals and three conditions: rest (black), agonist activation (striped) and antagonist activation (dotted). Compared to the condition specific baseline, the H-reflex was facilitated by TMS at rest at conditioning-test (C-T) intervals –2 to +18 ms and +58 ms, and during agonist activation at C-T intervals –4 to +7 ms. The H-reflex was near significantly suppressed at C-T interval –1 ms during antagonist activation. Compared to the antagonist activation condition, H-reflex facilitation was significantly stronger at rest at C-T intervals –2 ms and +1 ms (and nearly significant at +7 ms) and during agonist activation at C-T intervals –4 ms, –2 ms and +1 ms. The degree of facilitation between the rest and agonist activation conditions did not differ statistically. H-reflex amplitudes are presented as percentages of their condition-specific baselines. All P-values are corrected for multiple comparisons using Bonferroni correction, and statistical significance is defined at P < 0.05. Brackets indicate near-significant effects.

**Fig. 3:**
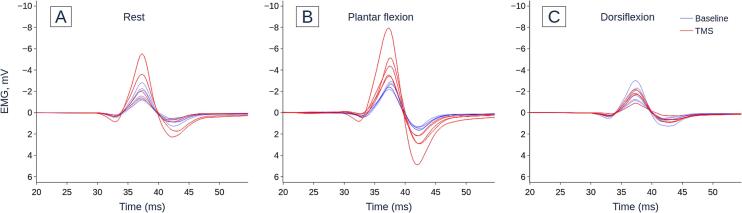
Representative H-reflex recordings of one subject during A) rest, B) plantar flexion and C) dorsiflexion conditions. Dotted blue line = baseline i.e. no TMS, solid red line = conditioning-test (C-T) interval +1, i.e. TMS preceded electrical stimulation by 1 ms. TMS enhances the H-reflex during the rest (A) and plantar flexion (B) conditions, but not during the dorsiflexion (C) condition. (For interpretation of the references to color in this figure legend, the reader is referred to the web version of this article.)

After individual latency correction we observed a statistically significant C-T interval effect in all measurement conditions (rest χ^2^(9) = 40.145, P < 0.001, agonist activation χ^2^(9) = 60.916, P < 0.001, and antagonist activation χ^2^(9) = 39.578, P < 0.001). *Post hoc* analyses adjusted for multiple comparisons with Bonferroni correction, revealed significant H-reflex facilitation with respect to condition-specific baseline at the following C-T intervals at rest: +1 ms (P = 0.045), +7 ms (P = 0.027), +18 ms (P = 0.036) and +58 ms (P = 0.027) and during agonist activation at C-T interval ranges: –4 ms (P = 0.027), –2 ms (P = 0.027), +1 ms (P = 0.045) and +7 ms (P = 0.027). Also rest –2 ms was close to significant (P = 0.054). During antagonistic activation, a near significant suppression of the H-reflex was observed at C-T interval –1 ms (P = 0.090) (see Fig. 2 and Fig. 3). Without using the latency correction, the results remained essentially the same, although during agonist activation the onset of H-reflex amplitude facilitation occurred slightly later (see Supplementary Fig. S1 for details). Age was not correlated with the magnitude of the modulation of any of the significant C-T intervals during the rest and plantar flexion conditions (Spearman’s Rho: during rest at C-T intervals +1 ms P = 0.580, +7 ms P = 0.750, +18 ms P = 0.593, +58 ms P = 0.631, and during plantar flexion at C-T intervals –4 ms P = 0.709, –2 ms P = 0.201, +1 ms P = 0.385 and +7 ms P = 0.555).

A slight decline in H-reflex facilitation was observed following the participant-specific first peak of facilitation at rest in 5/11 participants. This, however, remained statistically insignificant at group level, even after aligning the C-T intervals displaying the first peak of facilitation for each participant, and comparing this C-T interval to the following C-T interval 2 ms later (χ^2^(9) = 2.273, P = 0.132). Age did not differ between those participants presenting this slight decline in H-reflex facilitation, and those that did not (Mann Whitney U: P = 0.792).

### 3.2 Effect of voluntary muscle activation on the supraspinal modulation of the H-reflex

We found a statistically significant condition effect at C-T intervals –4 ms (χ^2^(2) = 8.909, P = 0.012), –2 ms (χ^2^(2) = 15.273, P < 0.001), +1 ms (χ^2^(2) = 15.800, P < 0.001) +7 ms (χ^2^(2) = 7.818, P = 0.020) and +18 ms (χ^2^(2) = 8.909, P = 0.012). *Post hoc* tests adjusted for multiple comparisons, showed that compared to the antagonist condition, H-reflexes were significantly enhanced in the agonist condition at C-T interval –4 ms (P = 0.03), in both the rest and agonist activation conditions at C-T intervals –2 ms (rest P = 0.012, agonist activation P = 0.009) and +1 ms (rest P = 0.015, agonist activation P = 0.015), and near significantly in the rest condition at C-T interval +7 ms (P = 0.078). No significant H-reflex amplitude differences were observed between the rest and agonist activation conditions. Fig. 2 summarizes the results. The main results did not differ significantly if latency correction was not used (refer to Supplementary Fig. S1).

## 4 Discussion

We employed the C-T paradigm to examine the temporal pattern of how sub-motor threshold TMS influences spinal excitability quantified using H-reflex recordings at rest and during voluntary muscle contractions in children and adolescents. We found that at rest, the supraspinal input facilitated the spinal excitability within two distinct periods: at corrected C-T intervals from +1 to +18 ms and again at C-T interval of +58 ms (C-T interval 0 ms reflecting the simultaneous activation of spinal neurons by central and peripheral volleys). A similar early enhancement of the H-reflex was observed during voluntary agonist activation (plantar flexion), with a slightly earlier onset (C-T interval –4 ms), but the delayed facilitation was absent. As expected, voluntary antagonist activation (dorsiflexion) counteracted the cortically induced facilitation, resulting in no early soleus H-reflex facilitation during dorsiflexion.

### 4.1 Correcting for individual differences in conduction latencies between peripheral and cortical volleys

The latencies of peripheral (H-reflex) and cortical (MEP) volleys were not equal, and these latency differences varied between participants. Therefore, we could only assume that the pre-determined C-T intervals reflected the time difference between the administration of peripheral and cortical stimuli, but not the timing of one volley entering the spinal cord with respect to the other. The exact arrival time of the supraspinal and peripheral volleys to the spinal cord is difficult to determine as their latencies are influenced by the number of synaptic connections in the pathway and the summation of indirect waves (I-waves) produced by the TMS pulse (Day et al., 1987, Sakai et al., 1997, Spampinato et al., 2023, Yu et al., 2024). As we were interested in the collision time of the two volleys in the spinal cord rather than the time at which stimulation was administered, we retrospectively adjusted the C-T intervals of each subject, so that C-T interval of 0 ms corresponded to the time when the two volleys are presumed to activate the spinal neurons simultaneously (refer to Methods). Such a correction has not been carried out in previous adult C-T studies, which is important to remember when comparing the results. Although our main results using the corrected data in children did not substantially differ from those obtained using the corresponding uncorrected data (refer to Supplementary Fig. S1), this correction ensures that modulatory changes in spinal excitability are accurately accounted for.

### 4.2 Tms-induced short-latency early facilitation appears pronounced in children at rest

In our study on children, sub-motor threshold TMS (intensity 90 % RMT) facilitated the soleus H-reflex significantly from C-T interval +1 onwards at rest, and a near significant facilitation was observed already at C-T –2 ms. In adults, a corresponding short-latency facilitation – linked to the activation of direct corticomotoneuronal connections (Nielsen et al., 1993, Nielsen and Petersen, 1995a, Xu et al., 2022) – has been observed at C-T intervals of –5 to –3 ms, but typically at supra-threshold TMS intensities or during voluntary muscle contractions (Cowan et al., 1986, Nielsen et al., 1993, Nielsen and Petersen, 1995a, Niemann et al., 2018, Andrews et al., 2020). Our finding, that TMS at sub-motor threshold intensity administered in a resting condition, was sufficient to enhance soleus H-reflex facilitation this early, suggests that lower motoneurons are more sensitive to supraspinal influence in children than in adults. Alternatively, the absence of a corresponding facilitation in adults at sub-motor threshold intensities at rest, could be due to uncorrected latency differences between afferent and efferent volleys, masking this relatively weak, short facilitation. However, we observed a similar early facilitation in children using both our uncorrected and corrected data (see Supplementary Fig. S1).

### 4.3 TMS-induced early suppression is diminished in children

We did not observe suppression of the soleus H-reflex amplitudes with respect to baseline in children at rest. In adults, an early inhibition effect at rest has been reported at C-T intervals of –3 to –1 ms, followed by a second inhibition at + 1 to + 4 ms (Nielsen et al., 1993, Nielsen and Petersen, 1995a, Andrews et al., 2020). Both occur with subthreshold TMS intensities of 65–95 % RMT for the first and 95 % RMT for the second (Andrews et al., 2020). The first inhibition is thought to be mediated by TMS-induced excitatory postsynaptic potentials acting on the reciprocal primary afferent interneurons in the spinal cord (Nielsen et al., 1993). The second, slightly delayed, inhibition is proposed to be mediated by an extrapyramidal pathway, potentially the corticoreticulospinal pathway, which inhibits lower motoneurons of the soleus in animals (Takakusaki et al., 1989). This pathway has a higher activation threshold and conducts more slowly than the corticospinal pathway (Andrews et al., 2020). We used sub-motor threshold TMS intensity of 90 % RMT, which is within the threshold range matching the earliest inhibition observed in adults but failed to observe an early inhibition of spinal excitability in children. A subset of the children in our study (5/11), however, displayed a slight non-significant decline in H-reflex amplitudes after an initial facilitation peak. The absence of an early inhibition in children may reflect immaturity of the developing spinal circuits and/or motor cortex, however, within our study group, age did not differ between those that presented the tendency, and those that did not. It is, however, possible that the number of subjects was too small to demonstrate a significant relationship with age.

### 4.4 TMS induces long-latency early facilitation in children at rest

In children, sub-motor threshold TMS enhanced spinal excitability continuously from C-T interval + 1 ms until C-T interval + 18 ms. We suggest that the early part of this facilitation reflects the short-latency early facilitation caused by the activation of direct corticomotoneurons as discussed above, while the latter part corresponds to long-latency early facilitation observed in adult studies at C-T intervals + 10 to + 20 ms (Nielsen et al., 1993, Guzmán-López et al., 2012; Guzmán-López et al., 2015; Niemann et al., 2018, Andrews et al., 2020, Xu et al., 2022). Long-latency early facilitation is thought to be generated by supraspinal input travelling via slower conducting corticospinal tract fibers to spinal cord interneuron and temporally summating (<20 ms) in alpha motoneurons (Valls-Sole et al., 1994, Goulart et al., 2000, Costa et al., 2011, Moini et al., 2024), depolarizing them and bringing them closer to their firing threshold. In addition, supraspinal volleys enhance spinal excitability by temporarily blocking presynaptic inhibition of Ia afferent terminals (Rudomin, 1990, Valls-Sole et al., 1994, Costa et al., 2011). It is likely that the same neuronal mechanisms explain the long-latency early facilitation of spinal networks in both children and adults.

### 4.5 A delayed re-facilitation effect is observed in children at rest

In children, we observed a delayed re-facilitation effect of the soleus H-reflex during rest at a corrected mean C-T interval of +58 ms. This delayed re-facilitation was separated from the early (short- and long-latency) facilitation by a period of no facilitation at a mean C-T interval latency of + 38 ms. In adults, delayed re-facilitation has been reported from C-T intervals +70 to +120 ms. It is preceded by a non-responsive period at C-T intervals +40 to +60 ms (Guzmán-López et al., 2012), reflecting the decaying of supraspinal-induced excitatory postsynaptic potentials and the restoration of presynaptic inhibition in the Ia afferents, and implies that the early and delayed facilitatory events are caused by distinct mechanisms. The mechanisms underlying the delayed re-facilitation (70–120 ms in adults) are not well understood. In adults, re-facilitation at latencies around +70 ms has been suggested to originate from descending slower conducting volleys such as indirect corticospinal or corticobulbospinal projections (Dimitrijević et al., 1992). Facilitation at longer latencies of up to +120 ms in adults, have been attributed to segmental re-afferentation volleys from cortically activated unmonitored muscles (Goulart et al., 2000, Costa et al., 2011, Guzmán-López et al., 2012). However, as our protocol did not extend beyond C-T intervals of +58 ms, we were unable to fully characterize the delayed re-facilitation phenomenon in children.

### 4.6 TMS-induced early facilitation during muscle activation in children

In children, the onset of the short-latency early facilitation elicited by TMS was observed at shorter C-T intervals during agonist activation (–4 ms) than at rest (+1 ms). It is likely that due to the preactivation of the motor cortex and the spinal motoneurons by voluntary muscle activation, fewer TMS-induced corticospinal volleys (I-waves) are required to activate spinal motoneurons, resulting in an earlier onset of facilitation of the alpha motoneuronal pool in the active state compared to rest (Lazzaro et al., 1998).

Contrary to what has been reported in adults, agonist muscle activation in children did not further enhance TMS-induced long-latency early facilitation. In adults, it is proposed that this increment in TMS-induced early facilitation is caused by lowered firing thresholds of spinal alpha motoneurons (Lazzaro et al., 1998, Nielsen and Petersen, 1995b), and stronger presynaptic inhibitions of spinal Ia inhibitory circuits (Nielsen and Kagamihara, 1993, Iles, 1996; Guzmán-López et al., 2015) during agonist activity. It is unclear why this effect was absent in children, but contributing factors may include differences in the way children and adults activate their muscles (i.e. muscle fiber composition and metabolic profile and activation of motor-units (Woods et al., 2022). For example, it has been proposed that children are less efficient than adults at recruiting higher-threshold (type II) motor units, leading to lower maximal neuromuscular activation in children (Dotan et al., 2012).

Antagonist activity (dorsiflexion) counteracted the TMS-induced facilitation of the soleus H-reflex in children, consistent with findings in adults (Guzmán-López et al., 2015). In adults, this has been attributed to reciprocal inhibition (Tanaka, 1974, Crone et al., 1987, Nielsen et al., 1993, Crone and Nielsen, 1994).

We did not observe a delayed re-facilitation effect during the active conditions. However, during both voluntary agonist and antagonist activation, we noted a trend toward increased amplitudes at our longest C-T interval + 58 ms. It is possible that we could have identified a delayed re-facilitatory effect during agonist and antagonist activation if we had included longer C-T intervals in our protocol.

## 5 Strengths and limitations

When using the H-reflex to assess the excitability of the motoneuronal pool, it is crucial to keep posture and muscle activity consistent throughout the measurement (Misiaszek, 2003, Schieppati, 1987). We achieved this by carefully monitoring limb position and by using a force transducer and providing visual feedback to the participant to ensure that a stable muscle force was maintained throughout the experiment.

Our sample size was relatively small given the broad age range (7–16 years), which limited our ability to analyze potential age-specific differences within the sample. Demonstrating maturational changes of inhibitory control over spinal networks would also have been more robust, had we been able to collect data from younger children (<7y) in our study. This study protocol was, however, not applicable to children younger than school age as it lasted two hours and required children to stay completely still repeatedly for the different measurements. It is, therefore, important to acknowledge that this study is unable to evaluate maturational changes occurring in the first seven years.

Additionally, restricting C-T intervals to eleven in our protocol did not enable a more detailed examination of the modulation time course. However, adding more C-T intervals would have significantly extended the measurement time, which was already two hours long, making it challenging for children to remain still and focused.

In our study on children and adolescents, we improved the accuracy of the C-T paradigm by retrospectively correcting for latency differences between the peripheral and cortical volleys for each participant, to better reflect the collision time of the volleys in the spinal cord. However, as fewer samples fell into the same C-T intervals, grouping and averaging of temporally adjacent intervals was necessary. Nevertheless, we feel that correcting for latency differences is a more accurate approach than leaving them uncorrected.

## 6 Conclusions

In children and adolescents, spinal excitability is enhanced by sub-motor threshold TMS of the motor cortex. Cortical modulation of spinal excitability through TMS appears to operate on a similar time scale as reported in adults, indicating functional similarities across both developing and mature nervous systems. However, notable differences exist, such as the lack of additional enhancement of TMS-induced early facilitation during voluntary agonist activation compared to resting conditions, likely due to the immaturity of the nervous system. Further research is needed to explore how cortical input affects spinal networks across different postures and during functional tasks in children. A deeper understanding of the fundamental mechanisms underlying motor control development may provide insight into motor control disturbances or spinal cord injuries that contribute to motor control challenges in the developing nervous system. This study lays the foundation for investigating the functional maturation of corticospinal excitability and spinal pathways involved in voluntary movement.

Author contributions

EMR Conceptualization, investigation, formal analysis, writing − original draft preparation. PN Conceptualization, investigation, formal analysis, review & editing, supervision. JT Investigation, review & editing. JGL Conceptualization, review & editing. HP Conceptualization, review & editing. LL Conceptualization, review & editing, supervision. All authors approved the final version of the manuscript.

Data availability

All data analyzed during this study are included in this published article (and its Supplementary Information file).

## Declaration of competing interest

The authors declare that they have no known competing financial interests or personal relationships that could have appeared to influence the work reported in this paper.

## Appendix A Supplementary data

Supplementary Figure 1 Supraspinal modulation of the soleus H-reflex amplitude using original conditional-test (C-T) intervals
