# Supplementary figures and images for: Spinal excitability is enhanced by transcranial magnetic stimulation of the motor cortex in children and adolescents
Source: Clin Neurophysiol Pract. 2025 Jul 6;10:278–85. doi: 10.1016/j.cnp.2025.06.009 (PMC12281449; doi:10.1016/j.cnp.2025.06.009)

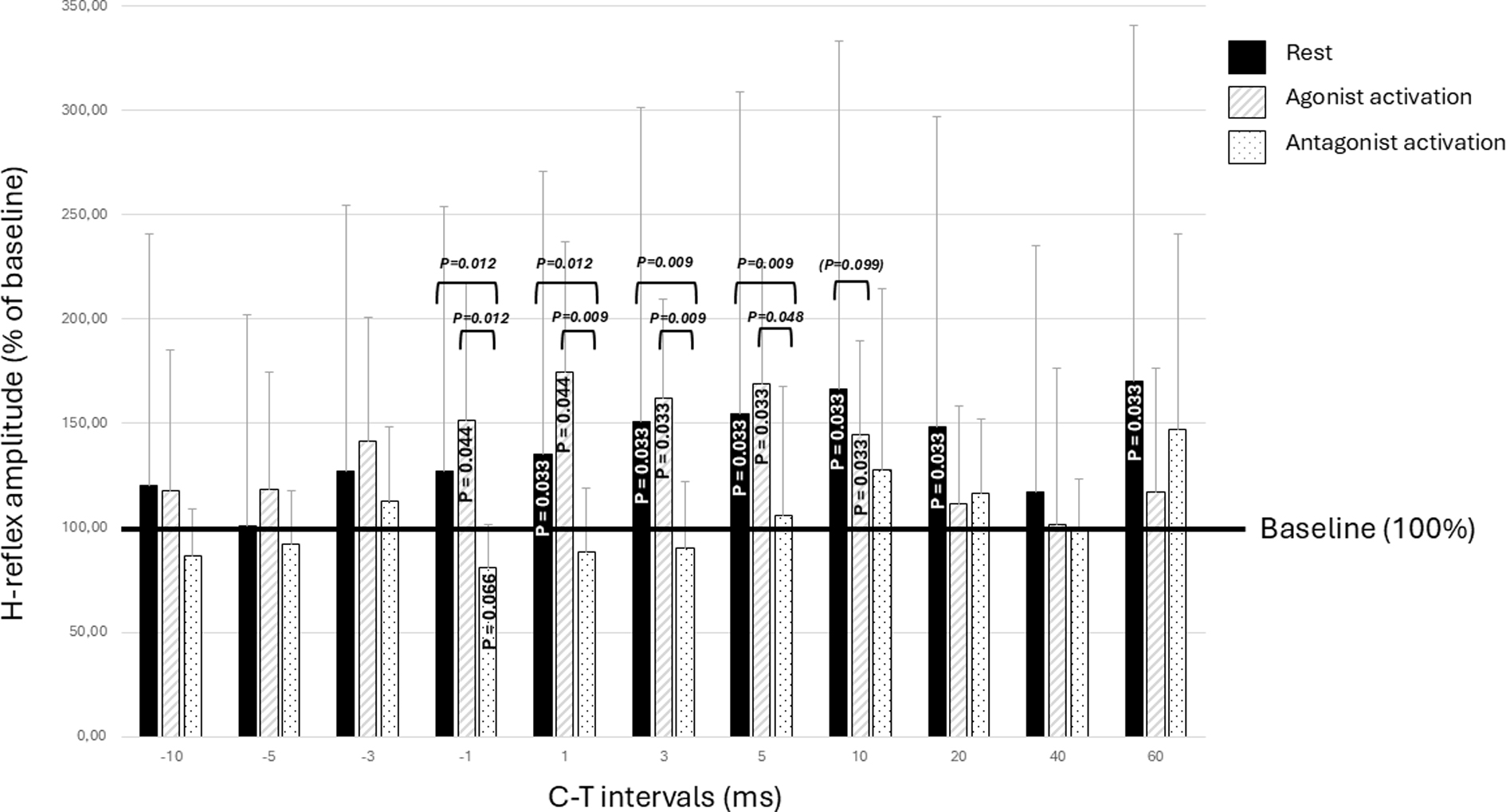

Supplement: Supplementary Figure 1 — Supraspinal modulation of the soleus H-reflex amplitude using original conditional-test (C-T) intervals [file mmc1.jpg]
